# Colon Bowel Preparation in the Era of Artificial Intelligence: Is There Potential for Enhancing Colon Bowel Cleansing?

**DOI:** 10.3390/medicina59101834

**Published:** 2023-10-15

**Authors:** Antonio Z Gimeno-García, Federica Benítez-Zafra, David Nicolás-Pérez, Manuel Hernández-Guerra

**Affiliations:** Gastroenterology Department, Hospital Universitario de Canarias, Instituto Universitario de Tecnologías Biomédicas (ITB) & Centro de Investigación Biomédica de Canarias (CIBICAN), Internal Medicine Department, Universidad de La Laguna, 38320 Tenerife, Spaindnicolasp@telefonica.net (D.N.-P.); mhernand@ull.edu.es (M.H.-G.)

**Keywords:** colonoscopy, bowel preparation, artificial intelligence, predictive scores, Boston bowel preparation scale

## Abstract

Background and Objectives: Proper bowel preparation is of paramount importance for enhancing adenoma detection rates and reducing postcolonoscopic colorectal cancer risk. Despite recommendations from gastroenterology societies regarding the optimal rates of successful bowel preparation, these guidelines are frequently unmet. Various approaches have been employed to enhance the rates of successful bowel preparation, yet the quality of cleansing remains suboptimal. Intensive bowel preparation techniques, supplementary administration of bowel solutions, and educational interventions aimed at improving patient adherence to instructions have been commonly utilized, particularly among patients at a high risk of inadequate bowel preparation. Expedited strategies conducted on the same day as the procedure have also been endorsed by scientific organizations. More recently, the utilization of artificial intelligence (AI) has emerged for the preprocedural detection of inadequate bowel preparation, holding the potential to guide the preparation process immediately preceding colonoscopy. This manuscript comprehensively reviews the current strategies employed to optimize bowel cleansing, with a specific focus on patients with elevated risks for inadequate bowel preparation. Additionally, the prospective role of AI in this context is thoroughly examined. Conclusions: While a majority of outpatients may achieve cleanliness with standard cleansing protocols, dealing with hard-to-prepare patients remains a challenge. Rescue strategies based on AI are promising, but such evidence remains limited. To ensure proper bowel cleansing, a combination of strategies should be performed.

## 1. Introduction

A colonoscopy is the gold standard for identifying colorectal neoplastic lesions. Its use in asymptomatic individuals enables the early detection of neoplastic lesions such as colorectal adenomas and early colorectal cancer (CRC) [1]. When used as a screening tool, a colonoscopy has the potential to decrease the incidence and mortality associated with CRC [2]. Enhanced colonoscopy efficiency is sought through proposed quality criteria, encompassing adherence to established indications and the recommended postpolypectomy surveillance intervals [3].

Of paramount significance are two pivotal indicators of quality: the cecal intubation rate and the adenoma detection rate, and both are intrinsically tied to proficient colon cleansing [3]. Inadequate cleansing decreases colonoscopy efficiency due to the necessity for repeat procedures, generating increased expenses [4]. Furthermore, it engenders delays in diagnosing malignant or precancerous lesions, curtails the adenoma detection rate (ADR), and increases procedural times and possibly patient risk. This predicament garners heightened importance within the prevailing backdrop of the COVID-19 crisis, wherein, for a substantial proportion of elective procedures, some endoscopy units have reported up to a 95% deferment of the endoscopy workload owing to the state of emergency [5]. The implications of such delays on patient prognoses remain uncertain.

Given these exigencies and the evolving landscape, the need for strategies for increasing colonoscopy efficiency is significant. Achieving proper bowel cleansing is of crucial importance for increasing efficiency. Acceptable benchmarks, deemed to fall within the 10% to 15% range, have been established [4,6]. However, the prevalence of suboptimal colonoscopies across endoscopy units evinces considerable variability in studies, spanning from 6.8% to 33% [7]. A multitude of factors have been linked to inadequate bowel preparation, prompting endeavors to mitigate suboptimal bowel cleansing through interventional studies aimed at high-risk patients with poor bowel cleansing [8].

In cases of low efficacy of the preparation protocol, strategies such as increasing the colon preparation volume or extending the duration of a low-fiber diet have been implemented [4,9]. Conversely, educational interventions could be useful when deficient adherence to preparation instructions surfaces as the chief concern [10]. As a final recourse, rescue cleansing strategies have been used [11]. Notably, recent advancements have incorporated artificial-intelligence-driven devices for guiding bowel preparation, and they have exhibited promising outcomes [12].

This manuscript provides a comprehensive review of the current recommendations, predictive factors for poor bowel cleansing, and current strategies to decrease inadequate bowel preparation.

## 2. Summary of the Current Recommendations

The current guidelines provide a set of general rules for patient preparation prior to a colonoscopy [4,6]. Timing is the most important factor to achieve proper bowel preparation [4,6]. Several meta-analyses have found a significant benefit of split-dose regimens (part of the bowel preparation administered the day before the colonoscopy and the other part administered the same day) compared with day-before bowel preparation in terms of colon cleansing quality, as well as willingness to repeat the same protocol in the future [13,14]. Several interventional studies have also found benefits when using split-dose regimens regarding tolerability and, more importantly, ADR and advanced ADR [15,16,17]). For afternoon colonoscopies, although split-dose regimens have achieved the same quality as full-dose morning preparation, patient tolerability and willingness appear to be higher in the latter [18]. Conversely, for morning colonoscopies, full-dose early morning preparation showed lower rates of patient compliance, tolerability, and willingness compared with split-dose regimens [19,20]. Another important aspect within the timing is the time elapsed between the last dose of the bowel preparation solution and the examination. A meta-analysis of randomized controlled trials (RCTs) showed that the major benefit in terms of bowel cleansing during a colonoscopy was an interval of 3 h, though this benefit progressively decreased after 4–5 h [14]. Other factors appear to be less important. Although, traditionally, a liquid diet has been recommended the day before a colonoscopy, a low-residue diet (LRD) is not inferior to a liquid diet in terms of colon cleansing and has been shown to increase patient tolerance and willingness to repeat the procedure in several meta-analyses [21,22]. However, recently, several studies have questioned the role of diet in the quality of bowel preparation before a colonoscopy. Gimeno et al. found in an RCT that the number of LRD days had no impact on cleansing quality (one LRD day was similar to three LRD days) [23]. Additionally, three other trials supported these results [24,25,26]. Verbal and written instructions have also been recommended in the published guidelines since they have been proven to achieve better bowel cleansing than either written or verbal instructions provided separately [4,6]. Less evidence exists regarding the use of adjuvants in such a way that current guidelines neither recommend them nor advise against their use. Bisacodyl and oral simethicone are the adjuvants with the greatest amount of evidence [27].

## 3. Type of Bowel Cleansing Solutions

Colon cleansing solutions can be classified according to their mechanism of action into osmotic or stimulant agents. Osmotic agents work by drawing water into the colon, such as polyethylene glycol (PEG), or by increasing intraluminal water through the removal of water from the intravascular space, such as the following hyperosmolar salts: sodium phosphate and citrate/magnesium oxide. On the other hand, stimulant agents, such as sodium picosulfate and bisacodyl, induce contraction of the colonic wall and promote the evacuation of the colon’s contents.

Traditionally, the most commonly used solutions have been based on high-volume PEG (3–4 L) [6,28]. However, 5 to 15% of patients do not complete this preparation due to the high volume and/or unpleasant taste and large volume [29]. Therefore, low-volume preparations have been developed, typically employing a combination of PEG (2 or 1 L) and an adjuvant such as ascorbic acid (ASC) or preparations based on a combination of sodium picosulfate and citrate/magnesium oxide (MCSP).

Several meta-analyses conducted on unselected populations have found no differences in colon cleansing quality between high-volume PEG-based preparations and low-volume preparations [30,31]. However, the use of low-volume preparations has been associated with better patient acceptance, compliance, and tolerance and lower rates of adverse effects. Some recent meta-analysis of RCTs have suggested that preparations based on 1 L PEG plus ASC are not inferior or even superior to other low-volume preparations (e.g., 2 L PEG plus ASC or MCSP) [32,33]. 

While all bowel solutions carry the potential risk for dehydration and electrolyte imbalances, isosmotic preparations, such as PEG, are theoretically associated with lower risk [4,6]. Hyperosmotic preparations may increase the risks, particularly in elderly individuals who often have more systemic comorbidities, as well as in patients with advanced heart failure and renal failure [4]. However, despite the theoretical safety of PEG solutions in these patient groups, strong supporting evidence is lacking. RCTs typically exclude patients with severe comorbidities, such as moderate or severe renal failure or New York Heart Association III or IV (NYHA) heart failure. Evidence of safety is often derived from case series or retrospective studies [34,35]. In retrospective studies that included patients with mild renal failure (>60 mL/min/1.73 m^2^), hyperosmotic PEG-based solutions were found to be safe [34,35], but there is a lack of evidence for patients with severe renal failure. Evidence is also limited for patients with advanced heart disease. Although isosmotic solutions could be considered in this context, there have been reported cases of worsened heart failure [36].

Nevertheless, current guidelines do not recommend hyperosmotic preparations (e.g., MCSP, PEG plus ASC, sodium phosphate, and oral sulfate) for patients with severe renal insufficiency (creatinine clearance < 30 mL/min) or congestive heart failure (NYHA III or IV) [4,6]. Isosmotic preparations should be prescribed in these cases. These recommendations apply particularly to non-PEG solutions such as MCSP or sodium phosphate due to the risk of magnesium toxicity and acute phosphate nephropathy. There is insufficient evidence to recommend a specific bowel solution for elderly patients and pregnant individuals, but PEG-based solutions could be considered as an option.

## 4. Assessment of Bowel Cleansing

Colon cleansing should be evaluated after washing and aspirating all existing fecal residue. In practice, colonic cleansing is deemed adequate if it allows for the visualization of colorectal neoplastic lesions larger than 5 mm as these are considered clinically significant [6]. Conversely, an examination should be repeated within a period of one year if lesions of this size cannot be ruled out [4]. Currently, the following four rating scales for bowel cleansing have been widely studied and demonstrate sufficient validity and reliability: the Boston Bowel Preparation Scale (BBPS), the Ottawa scale, the Aronchick scale, and the Harefield scale [37,38]. A systematic review concluded that the BBPS provides the highest intra- and inter-observer agreement and the best correlation with the ADR, recommending its use in clinical practice [39]. According to the BBPS, a global score of ≥6, with segmental colonic scores of ≥2, ensures adequate colonic cleansing quality and allows medical practitioners to follow the recommended endoscopic surveillance intervals. As mentioned above, clinical societies recommend that the percentage of colonoscopies with inadequate colonic cleansing performed in an endoscopy unit should not exceed 10–15% per year, suggesting an audit if these figures are exceeded [39].

## 5. Predictors of Poor Bowel Cleansing

Although a majority of patients who undergo colonoscopies have adequate bowel preparation, inadequate cleansing occurs in 9–30% of colonoscopies [7]. Predictors of poor bowel cleansing can be classified as factors related to the bowel preparation protocol (as mentioned above, primarily timing), bowel preparation efficacy factors, and incompliance with the recommended instructions. Bowel preparation efficacy factors are associated with the inhibition of colonic motility, such as chronic constipation, abdominal or pelvic surgeries (especially in patients who have undergone with left colectomies), and the use of calcium antagonists, tricyclic antidepressants, opioids, as well as comorbidities (particularly diabetes mellitus), a high body mass index, and hospitalization [4]. Up to 20% of patients with inadequate colonic cleansing do not follow instructions [8]. Incompliance can be conscious or unconscious, and there may be different reasons, such as forgetfulness or living far from the hospital. Low tolerance is another reason for noncompliance. A recent meta-analysis demonstrated that sociodemographic characteristics (e.g., sex and age) are predictors of colon cleansing, with marginal effects, while comorbidities such as diabetes, stroke, or dementia and treatments such as opioids and tricyclic antidepressants are stronger predictors [40]. Several studies have found that the accumulation of various factors increases the likelihood of inadequate cleansing [41,42,43,44]. Four predictive models designed to detect patients with high risks for poor bowel preparation have been published thus far (Table 1) [41,42,43,44]. Hassan et al. conducted a prospective and multicenter study, recruiting 2811 consecutive patients for colonoscopies, where 33% of the patients had inadequate colon cleansing [44]. In this study, male sex, high body mass index, advanced age, previous colorectal surgery, hepatic cirrhosis, Parkinson’s disease, and diabetes mellitus were independent predictors of poor bowel cleansing. A positive fecal occult blood test was associated with adequate bowel preparation. The predictive model designed with these variables had a low discriminatory capacity (area under the curve (AUC): 0.63; 95% CI: 0.62–0.66). In a posterior study, Dik et al., in a prospective, multicenter study that included 1996 patients, designed another predictive model [42]. Predictors associated with this condition included a score of ≥3 for the American Society of Anesthesiologists Physical Status Classification System (ASA), the use of tricyclic antidepressants and opioids, diabetes, chronic constipation, a history of abdominal or pelvic surgery, previous inadequate colon cleansing, and hospitalization. In a retrospective single-center study, Berger et al. found that diabetes; cirrhosis; obesity; treatment with opioids, antidepressants, or neuroleptics; irregular physical activity; and abdominal surgery were significantly associated with poor bowel cleansing [41]. These models have significant methodological limitations, such as the inclusion of patients with histories of inadequate colon preparation, which is relevant for the following reasons: these patients could have benefited from intensive cleansing protocols, there was a lack of standardization in the type of colon cleansing solution used, there was an absence of split-dose regimens, the assessments of colon cleansing used non-validated scales, and they used retrospective designs. To overcome these limitations, Gimeno-García et al. analyzed predictors of inadequate colon cleansing in 1057 ambulatory patients that were prepared on the same day of examination with low- and high-volume colon cleansing solutions [43]. Cleansing was evaluated using the BBPS scale. Comorbidities, antidepressant use, chronic constipation, and pelvic or abdominal surgeries were independent predictors of inadequate colon preparation.

Overall, these models have modest accuracy (Table 1), and they are better at excluding poor bowel cleansing than they are at confirming it (i.e., they have higher negative predictive value than positive predictive value).

Recently, the characteristics of the last rectal effluent during bowel preparation were shown to be a good predictor of bowel cleansing during a colonoscopy, and they have been proposed for guiding bowel preparation before an examination [45,46]. In one prospective study, 633 consecutive outpatients were asked to choose a drawing (from a set of four drawings resembling different cleansing qualities) that most resembled their last stool during the bowel preparation [46]. Overall, 107 patients (16.9%) had inadequate cleansing during their colonoscopies. The patients’ perceptions had positive and negative predictive values of 54.6% and 88.3%, respectively. The agreement between patient perception and the BBPS was fair, although it was statistically significant (Cohen’s kappa coefficient of 0.37; *p* < 0.001). The results were similar in a validation cohort (Cohen’s kappa coefficient of 0.41).

## 6. Strategies for Improving Bowel Cleansing

The selection of a bowel-cleansing strategy depends on the cause of a failed bowel preparation. Therefore, two groups of patients can be differentiated. First, in patients with previous colonoscopies with poor preparation, efforts should be made to determine the cause of the failed bowel preparation because the strategy to follow will be different. In the case of poor tolerance to the bowel solution or incompliance, enhanced instructions (education) and/or providing the same tips to increase tolerance should be the strategy of choice. Conversely, if a patient followed instructions and their tolerance was good, the patient could benefit from intensive bowel preparations (typically accomplished by increasing the preparation volume). The other group of patients are those attending a first-time colonoscopy, and in these cases, the best approach is likely a combination of strategies (enhanced instructions and tips), and if we expect that such a patient has a high risk of failure (associated with the inhibition of colon motility) based, for example, on the aforementioned predictive scores, then intensive preparations could be the best strategy.

**Figure 1:**
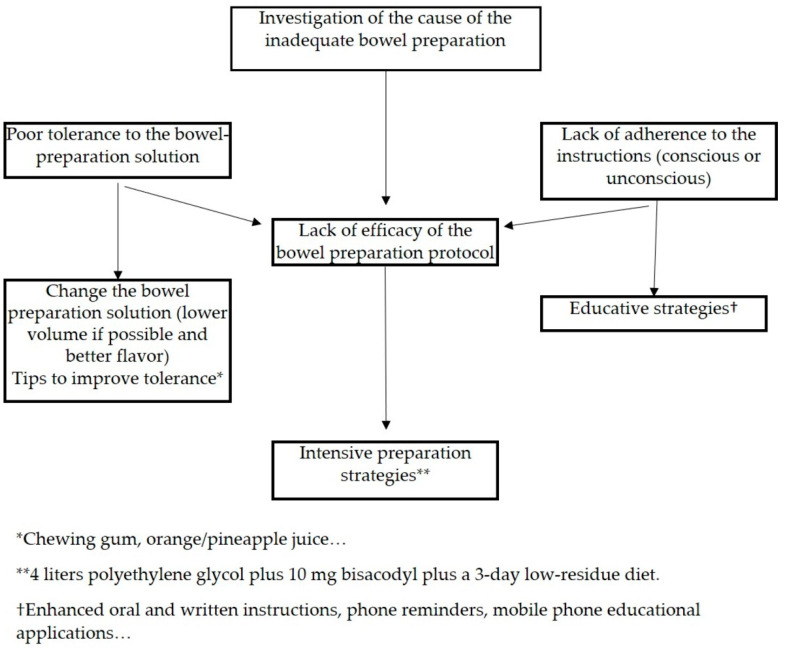
Proposal for improving bowel cleaning in patients with previous colonoscopies with inadequate bowel preparation.

**Figure 2:**
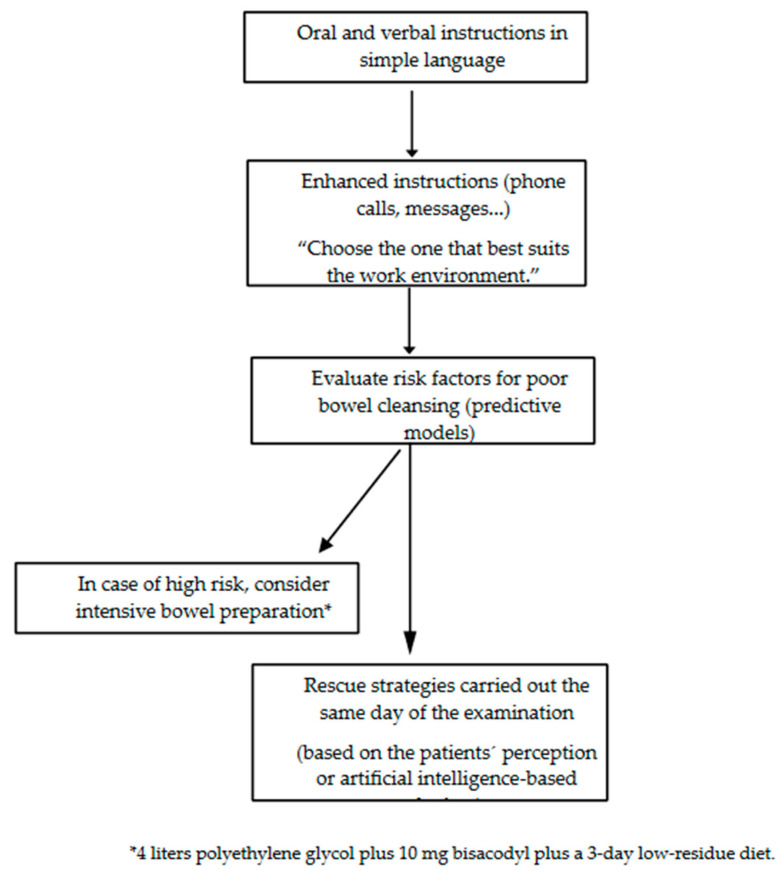
Tips for improving bowel cleaning in naïve patients.

A proposal for this approach is shown in Figure 1 and Figure 2.

Different strategies are set out in the next section.

### 6.1. Strategies for Increasing Tolerance

Different adjuvants have been recommended to improve palatability. Chewing gum has been proven to increase patients’ satisfaction and willingness to repeat the same bowel preparation and to reduce the time taken to drink the bowel preparation in RCTs. Chewing gum also reduces abdominal discomfort, nausea, and vomiting. Contradictory results have been found in terms of bowel cleansing [47,48]. Some beverages, either as bowel prep diluents, ingested during the pause interval of drinking the preparation, kept in the mouth before bowel preparation ingestion, or drunk after bowel cleansing, were tested [49,50,51]. In RCTs, Coca Cola used as a diluent has resulted in an improvement in the flavor, a shorter time to drink the bowel solution, increased willingness to repeat the same bowel preparation, and an increased quality of bowel cleansing during the colonoscopy [51]. Although other beverages have improved patient satisfaction and decreased side effects, they did not improve bowel cleansing [49,50]. A recent meta-analysis tested whether the use of adjuvants for improving palatability improved patient experience and increased cleansing quality. A total of six RCTs (with 1187 patients) using different adjuvants were included. Overall, the authors found that the adjuvants had significant benefits in improving the flavor and patient willingness to repeat the same bowel preparation protocol, and they decreased side effects and even improved the cleansing quality (odds ratio of 2.52, 95% confidence interval [1.31–4.85]) [52].

### 6.2. Strategies for Decreasing Incompliance

Incompliance has been stated as a major burden for bowel preparation. In a prospective study carried out on 462 outpatients, the probability of poor bowel preparation was more than twice as high in patients with poor adherence to the instructions. Incompliance was the most important predictor of poor bowel cleansing [53].

Several meta-analyses have been recently reported, highlighting the benefits of enhanced instructions in bowel cleansing [54,55,56,57]. The meta-analysis by Guo et al. also found a higher detection rate of polyps and adenomas in the group of patients who received the intervention. Generally, in these meta-analyses, compliance with the instructions, willingness to repeat the same preparation, and cleansing quality during the colonoscopy were higher in the groups of patients who received the educational intervention [56]. However, educational strategies encompass a heterogeneous group of interventions that may yield disparate results, leading to a lack of consensus on which intervention is the most effective. The types of educational interventions are discussed below based on the type of strategy employed:

- Individual or group informative sessions: These sessions are conducted by trained health care personnel, and in them, a patient receives instructions regarding dietary aspects, the type and administration of the evacuating solution, and precautions to be taken with the home treatment. The results are conflicting in the published studies [58,59].
- Printed educational materials: Using brochures or pamphlets that combine text with illustrative images or drawings about good or poor colon cleansing, lesions were detected based on colonic cleanliness and permitted or prohibited foods. The distribution of this material had positive effects on cleansing quality in most of the studies [60,61].
- Audiovisual material: Educational videos can enhance understanding through the use of simple words, illustrations, and video clips. Some RCTs have compared this strategy to the standard practice, with two studies observing better colon cleansing quality in the intervention groups [62].
- Phone calls or text messages: Through telephone communication, the importance of colonic preparation, the method of following the diet, and taking the evacuating solution are emphasized, along with addressing doubts and providing reminders of scheduled appointments. Such RCTs have demonstrated better colonic cleansing quality in patients assigned to intervention groups [63].
- Mobile applications and social networks: Mobile phones and social networks have become significant sources of medical information. RCTs have evaluated colon cleansing quality in patients who used smartphone applications detailing the information about the colonoscopy preparation, with explanatory images and/or videos, compared to the utility of receiving oral and written instructions [64]. Colonic cleansing quality was superior in the intervention groups in these studies [64,65].

### 6.3. Intensified Interventions

Once incompliance and a lack of tolerance have been excluded or we have already implemented interventions in high-risk patients to control these two factors, the third leg of the chair is to recommend enhanced bowel preparations. Several studies have compared low-volume-based preparations (1 L or 2 L of cleansing solution with or without an adjuvant) with high-volume-based preparations (3 L or 4 L of bowel preparation); however, few studies have compared intensified bowel protocols [66]. It makes sense to use this approach in patients with risk factors for poor bowel cleansing since a majority of patients will be cleansed with a standard bowel preparation. One RCT compared a high-volume-based enhanced bowel protocol (4 L of PEG plus bisacodyl) with a low-volume protocol (2 L of PEG plus ASC acid plus bisacodyl) in 256 patients who had failed standard bowel preparation [66]. The intensified high-volume protocol was superior in terms of cleansing quality in both the intention-to-treat analysis (odds ratio of 2.07, 95% confidence interval [1.16–3.69]) and in the per-protocol analysis (odds ratio of 2.55, 95% confidence interval [1.4–32.92]). Interestingly, the patients who benefited most from the high-volume protocol were those who received a standard low-volume preparation solution in their first colonoscopies. No statistically significant differences were found regarding tolerance or ADR between the groups.

In patients undergoing first-time colonoscopies, one RCT compared an enhanced bowel preparation protocol (4 L of PEG plus bisacodyl) with a standard low-volume protocol (2 L of PEG plus ASC) in 260 patients with high risks of poor bowel preparation. These patients were selected by using a predictive score [23]. In this study, an enhanced high-volume preparation protocol was not better than the standard protocol either in the intention-to-treat analysis (ITT) or in the per-protocol analysis (PP). The authors hypothesized that the low sample size could have hindered the results of the study since in a majority of the patients, administering a conventional colon preparation would have been sufficient.

Therefore, the implementation of enhanced bowel preparations remains controversial.

### 6.4. Rescue Strategies

Same-day or next-day colonoscopies after additional bowel preparation have been suggested in the current guidelines, although with a low level of evidence and a low grade of recommendation [4]. There are some experiences using this type of strategy. Yang et al. randomized 131 patients with poor bowel preparation (BBPS of < 2 in at least one segment) to a 1 L PEG enema administered through the colonoscopy channel in the right colon or 2 L of oral PEG plus ascorbic acid during same day of the examination [11]. The additional oral solution managed to rescue up to 81% of the patients compared to 50% with the enema administration (*p* < 0.001). Different devices based on endoscopic irrigation pumps have been developed [67,68,69]. These devices utilize pressurized water, saline solutions, or even CO2 combined with a suction system, and these are introduced through the working channel of the endoscope or in parallel to it, or they are used prior to the procedure. In general, and in the absence of randomized studies with larger sample sizes, these devices appear to enhance the quality of colon cleansing.

## 7. Role of Artificial Intelligence in Improving Bowel Cleansing

At present, a notable advancement is unfolding in the realm of artificial intelligence (AI) applications within the medical field, specifically in gastroenterology and especially in gastrointestinal endoscopy [70]. Concerning bowel preparations, a majority of the research efforts have focused on training and verifying convolutional neural networks (CNNs) to identify bowel cleanliness levels during colonoscopies utilizing well-established cleansing scales [71]. These systems hold promise in overcoming the difficulties arising from discrepancies in assessments between various observers while evaluating colon cleanliness during colonoscopy procedures. Within this array of systems, ENDOANGEL has emerged as the only commercially accessible real-time solution for assessing colon cleanliness [72]. Although these systems are useful for more objectively assessing bowel cleansing during a colonoscopy, they do not help to prevent poor bowel preparation.

Recently, two RCTs carried out on Chinese populations utilized AI platforms using a CNN to guide bowel preparation. In both of these studies, the CNNs were trained with annotated images of rectal effluents [73,74].

In the first study, the CNN was trained using 4302 images, and it demonstrated excellent accuracy in predicting bowel cleansing (>95%) [73]. A total of 1454 patients were enrolled and randomized into either an intervention group or a control group. The patients in the intervention group were required to scan a quick response code (QR code) and upload an image of their rectal effluent during the bowel preparation process. The uploaded image was then analyzed by the CNN, providing an assessment of “pass” or “not pass”. For cases where the assessment was “not pass,” the system provided general instructions for enhancing the bowel preparations. The quality of bowel preparation, as assessed by the Boston Bowel Preparation Scale (BBPS), did not differ significantly between the groups (AI group: 90.7% vs. control group: 91.5%; *p* = 0.429). Upon comparing the CNN predictions with the BBPS, it became evident that the CNN model faced challenges in effectively discerning cleansing quality. Among the patients with BBPS scores of <6 points, only 6 out of 71 (8.45%) were correctly classified by the CNN, and the remaining images were erroneously classified as having sufficient preparation. Conversely, 26 patients with BBPS scores of ≥6 points were mistakenly categorized as having inadequate preparation. Consequently, the clinical utility of this CNN model could be limited when guiding intervention strategies for patients predicted to have poor cleansing quality on the same day. In the second study, a ShuffleNet v2 CNN was trained using 5362 images of rectal effluents, and it achieved an accuracy of 95.15% in predicting bowel cleansing quality [74]. Subsequently, a total of 524 patients were randomized to either a CNN-powered smartphone application or a control group. In this case, the intervention group demonstrated superior colonic cleansing quality compared to the control group, as evidenced by both the ITT analysis (88.54% vs. 65.59%, respectively; *p* < 0.001) and the PP analysis (89.78% vs. 65.59%, respectively; *p* < 0.001). Nevertheless, the rate of acceptable colon cleansing in the control group was significantly lower than anticipated (88.54% in the AI group vs. 65.59% in the control group).

These studies present promising results for guiding colon bowel cleansing on the same day as an examination. However, further research is warranted, especially in Western countries where the populations are aging and possess less familiarity with new technologies.

## 8. Future Directions

The best approach and protocol for colon-cleansing preparation remain unknown. They should achieve proper bowel preparation in a majority of the patients who are difficult to prepare. Customized protocols should be used when the cause of poor bowel preparation is already known, such as in patients with a history of inadequate bowel preparation. For these patients, the cause should be investigated and tailored strategies should be adopted. Interventions based on predictive scores do not appear to be very effective thus far possibly because these scores are not accurate enough to identify patients who are difficult to prepare. Thus, new and more accurate scoring systems need to be implemented in clinical practice.

Rescue strategies are the last option for enhancing bowel cleansing. Ideally, they should be applied before conducting a colonoscopy. In this way, a patient’s perception of their own bowel preparation quality before an examination—and before employing AI systems to assess the patients’ cleansing before the examination—could guide the next steps to follow. However, some concerns persist regarding these new technologies. First, they have only been tested in the Asian population and have conflicting results. They should be validated using Western populations. Second, there are concerns about the applicability of these new systems in different scenarios. They should also be simple enough to be used by patients with varying levels of technological knowledge. The progressive aging of Western populations is a factor that could hinder the efficiency of these new technologies. Additionally, these aging populations tend to have more comorbidities, making them the group of patients facing the most difficulties in achieving proper colon preparation. Studies combining many different strategies are needed to improve the efficiency of colonoscopies.

## 9. Conclusions

Achieving a proper colon cleansing quality is of paramount importance as it is closely related to the early detection of colorectal neoplasms and, consequently, the risk of interval colorectal cancer. Despite advancements made over the past few decades, quality standards are not frequently met. While a majority of outpatients may achieve cleanliness with standard cleansing protocols, dealing with hard-to-prepare patients remains a challenge. Interventional strategies involving intensive bowel preparations and educational approaches have been implemented, with conflicting results. Rescue strategies have the potential to enhance bowel cleansing. Recently, strategies guided by artificial intelligence that are applied on the same day as a colonoscopy have shown promise in improving bowel preparation, although such evidence remains limited. To ensure optimal preparation, it would appear logical to utilize a combination of all the available strategies at our disposal, particularly for high-risk individuals.

## Figures and Tables

**Table 1:** Current predictive models in outpatients.

	Hassan et al.	Dik et al.	Gimeno et al.	Berger et al.
Variables	- Obesity - Male sex - Age - Colorectal surgery - Cirrhosis - Parkinson’s disease - Diabetes - Positive FOBT *	- ASA ** score - Tricyclic antidepressants - Opioids - Diabetes - Chronic constipation - Abdominal/pelvic surgery - Hospitalization - History of inadequate preparation	- Tricyclic antidepressants - Comorbidities - Chronic constipation - Abdominal/pelvic surgery	- Diabetes/obesity - Irregular physical activity - Cirrhosis - Antidepressants/neuroleptics - Opioids - Abdominal surgery - History of inadequate preparation
AUC †, 95% CI	0.63	0.72–0.77	0.72–0.70	0.622–0.621
Sensitivity (%)	60	66	50	46
Specificity (%)	59	79	80	76
NPV ‡ (%)	41	29	36	39
PPV # (%)	76	95	88	81

*, fecal occult blood test; **, American Society of Anesthesiologists Physical Status Classification System; †, AUC in the development and validation cohorts; ‡, negative predictive value; #, positive predictive value.

## Data Availability

Data sharing is not applicable to this study.

## Abbreviations

CRC, colorectal cancer; ADR, adenoma detection rate; RCT, randomized controlled trial; LRD, low-residue diet; PEG, polyethylene glycol; ASC, ascorbic acid; MCSP, sodium picosulfate and citrate/magnesium oxide; NYHA, New York Heart Association III or IV; BBPS, Boston Bowel Preparation Scale; AUC, area under the curve; ASA, American Society of Anesthesiologists Physical Status Classification System; NPV, negative predictive value; PPV, positive predictive value; ITT, intention to treat analysis; PP, per protocol analysis; AI, artificial intelligence; CNN, convolutional neural network; QR code, quick response code.
